# Terahertz Biaxial Strain Sensor Based on Double-Upright Cross Metamaterial

**DOI:** 10.3390/mi14040816

**Published:** 2023-04-04

**Authors:** Yanfei Liu, Yu Chen, Jing Li, Chunli Zhang, Qiannan Wu, Ningning Su, Mengwei Li

**Affiliations:** 1 School of Semiconductors and Physics, North University of China, Taiyuan 030051, China; 2 Center for Microsystem Integration, North University of China, Taiyuan 030051, China; 3 School of Instrument and Intelligent Future Technology, North University of China, Taiyuan 030051, China; 4 Academy for Advanced Interdisciplinary Research, North University of China, Taiyuan 030051, China; 5 School of Instrument and Electronics, North University of China, Taiyuan 030051, China; 6 Key Laboratory of Dynamic Measurement Technology, North University of China, Taiyuan 030051, China

**Keywords:** terahertz, metamaterial, biaxial strain, pressure sensor

## Abstract

In this article, a terahertz metamaterial biaxial strain pressure sensor structure is proposed, which can address the problems of the low sensitivity, the narrow pressure measurement range, and the uniaxial-only detection of existing terahertz pressure sensors. The performance of the pressure sensor was studied and analyzed using the time-domain finite-element-difference method. By changing the substrate material and optimizing the structure of the top cell, the size of the structure that can simultaneously improve the range and sensitivity of the pressure measurements was determined. The simulation results show that the sensor has a pressure-sensing effect in the frequency range of 1.0–2.2 THz under the conditions of transverse electric (TE) and transverse magnetic (TM) polarization, and the sensitivity can reach up to 346 GHz/μm. The proposed metamaterial pressure sensor has significant applications in the remote monitoring of target structure deformation.

## 1. Introduction

Terahertz (THz) waves are electromagnetic waves with a frequency range of 0.1–10 THz [1]. A THz has the advantages of a wide frequency range, strong penetration, and low energy, and is applied in medical imaging, wireless broadband communication, cosmic astronomy, sensor detection, and other research fields [2,3]. However, ensuring extreme sensitivity in sensor detection is challenging, due to the limited number of samples. To better probe the microscope, THz waves need to be locally enhanced, so various materials are considered from time to time.

Metamaterials are artificially designed, uniform, and electromagnetic materials with a periodic unit structure [4]. Due to their excellent properties, compared with conventional materials, they have been gradually applied by scientific researchers in the field of sensing, such as strain sensors [5], temperature sensors [6], imaging sensors [7], absorber sensors [8], refractive index sensors [9], and biosensors [10].

In 2021, Hamouleh-Alipou et al. presented a numerical method to demonstrate graphene-tunable metasurface sensors that were capable of excellent optical properties over a wide range of frequencies, with unique control and exploitation of light [11]. Current thermoelectric graphene-based metasurface structures are available for surface plasmon excitations in nanosensor platforms.

Another hypersurface biosensor was proposed in 2022 by Hamouleh-Alipou et al. [12]. The proposed optical hypersurface design can be used as a biosensor to detect the content of different hemoglobin concentrations in the whole blood by the sensor, making it one of the best candidates for future optical biosensors in biomedical applications. The electromagnetic wave can be regulated to a certain extent by changing the parameters of the resonant unit [13]. When external stresses, temperature, refractive index, phase, and other parameters are modified, the coupling properties of the coupling of a subwavelength periodic unit cell at the surface of the material to electromagnetic waves of a particular frequency are shifted. At this point, the dependence of the external parameters on the resonant frequency of the electromagnetic wave can be used to implement the sensing and detection functions.

In sensing and detection, pressure sensors account for 20% of the world’s market demand due to their strong practicality and wide range of applications. Different types of flexible pressure sensors have been reported, including piezoresistive [14] and piezoelectric [15]. Piezoresistive pressure sensors convert an external stimulus into a resistive shift of the active material, which works by inducing a shift in the contact resistance or conductance pathway between the active materials by applying pressure. For some anisotropic crystalline materials, the piezoelectric effect occurs during mechanical deformation, leading to a polarization of the internal dipole and, hence, a potential shift in the two converse faces of the crystal. 

In recent years, most pressure sensors have adopted wired measurements. However, these pressure sensors suffer from excessive power consumption, a low signal-to-noise ratio of the output signal, and poor anti-jamming capability. Therefore, their applications are limited to special contexts. Wireless passive pressure sensors with elevated stability, strong repeatability, high accuracy, and elevated resolution have gradually become the focus of research. 

In 2009, Melk et al. designed a strain sensor based on an open resonant ring structure on a silicon substrate, with an operating frequency of 12–13.1 GHz and a sensitivity of 0.292 MHz/kgf [16]. In 2011, Li et al. designed an I-shape structure with a sensitivity of 107 GHz/um in the range of 0.4–1.6 THz [17]. In 2015, Wei designed a cross shape with a sensitivity of 0.7 nm/% between 700–1000 nm wavelengths [18]; In 2017, Schuster et al. proposed a dielectric resonator structure, which can achieve a sensitivity of 3.7 MHz/KPa in the range of 17–21.4 GHz [19]. In 2020, Jeong et al. demonstrated a 3D hinged structure with a base whose sensor sensitivity was 1.132 × 10^5^ KHz/KPa in the 4.8–5.6 GHz operating frequency band [20].

Most of the reported metamaterial pressure sensors can only detect stress in a single polarization state and are concentrated in the microwave frequency band, while few studies have been performed in the THz band, where the sensitivity is low, the pressure measurement range is modest, and the applications are limited. Therefore, the study of biaxial strain THz material pressure sensors is of great significance to promote the development of high-frequency passive pressure-sensing technologies.

In this paper, we designed a biaxial pressure sensor structure based on THz metamaterials. Metamaterial structures of the extremely symmetric electromagnetic resonance type were adopted as a whole. The structure was able to simultaneously generate two modes of NMR in a terahertz frequency band, enabling biaxial stress detection as a function of different polarization states. The designed sensor provides a suitable design scheme for detecting object deformations in special environments.

## 2. Structure Design

**Figure 1:**
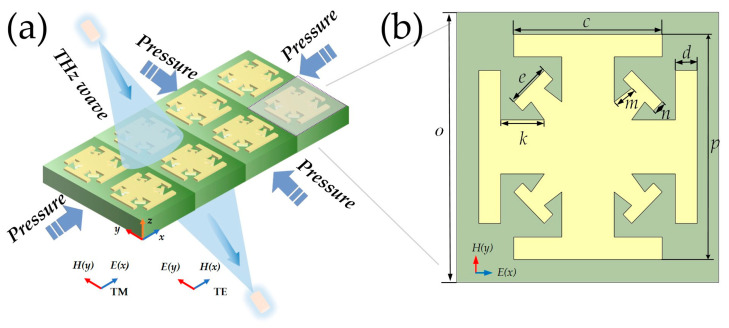
Metamaterial pressure sensor: (**a**) overall structure drawing; (**b**) view of metamaterial unit.

In this paper, we designed a two-layer metamaterial THz pressure sensor. The periodic structure diagram is shown in Figure 1a. Periodic boundary conditions for the structural unit cell along the *X*-direction are shown in Figure 1b. The sensor was a double-layer structure with two identical double-upright cross units on top. The material was gold (Au), the conductivity was 4.56 × 10^7^ S/m, and the thickness was 0.2 um. The substrate was a lossless dielectric material Teflon with a thickness of 100 um and a dielectric constant of 1.84. The spacing between two double-upright cross font structures was defined in this paper as g=o−p.

The detailed parameters of the optimized single double-upright cross structure of the metamaterial pressure sensor are shown in Table 1. The simulation analysis of the sensor in the TM and TE polarization states was performed using the time-domain finite-element-difference method.

## 3. Simulation Results and Analysis

**Figure 2:**
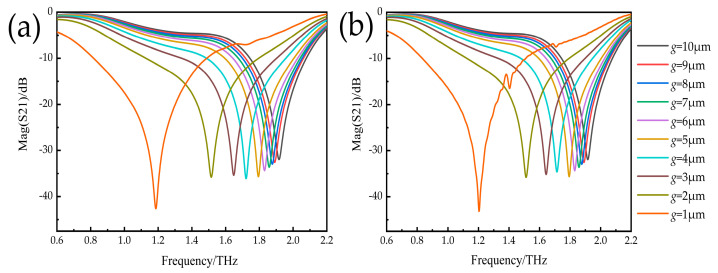
Transmission coefficient spectrum diagram: (**a**) TM incident S21 spectrum diagram; (**b**) TE incident S21 spectrum diagram.

In this paper, the time-domain solver of CST Microwave Studio was used to simulate the pressure sensor. The electromagnetic wave was perpendicular to the surface of the structure. Stress simulations were performed on the *Y* and *X* axes of the sensor in the TM and TE polarization states, respectively. The spectrum diagram of the transmission coefficient (S21) is shown in Figure 2.

When the incident THz wave was set in the TE polarization state, pressure deformation was applied to the sensor in the *Y*-axis direction. The results of the simulation are shown in Figure 2a. The PTFE substrate was elastic, except for the metal-covered area, and compression of the pressure sensor resulted in a smaller spacing between the two double-upright cross structures. When the compression amount of the electromagnetic pressure sensor was 0, the corresponding resonant frequency was 1.92 THz. The resonant frequency changed from 1.92 THz to 1.91 THz when the spacing of resonant cells changed from 10 μm to 9 μm and the compression was 1 μm. When compressed from 9 μm to 8 μm, the resonant frequency changed from 1.91 THz to 1.89 THz. When compressed from 8 μm to 7 μm, the resonant frequency changed from 1.89 THz to 1.87 THz; When changing from 7 μm to 6 μm, the resonant frequency changed from 1.87 THz to 1.84 THz. When changing from 6 μm to 5 μm, the resonant frequency changed from 1.84 THz to 1.78 THz. When changing from 5 μm to 4 μm, the resonant frequency changed from 1.78 THz to 1.73 THz. When changing from 4 μm to 3 μm, the resonant frequency changed from 1.73 THz to 1.65 THz. When changing from 3 μm to 2 μm, the harmonic frequency changed from 1.65 THz to 1.52 THz. When changing from 2 μm to 1 μm, the resonant frequency changed from 1.52 THz to 1.17 THz. The variations in the resonant frequency of the pressure sensor as the spacing decreased is shown in Table 2.

The simulation results showed that when pressure was applied to the sensor, the surface gap decreased gradually, the resonant frequency moved from 1.92 THz to 1.17 THz, and the resonant frequency changed from 10 GHz/μm to 750 GHz/μm. As the gap width decreased, the capacitance in the gap increased, resulting in a blueshift in the resonance frequency, with a maximum frequency shift of 46.6% per micron. The simulation results are shown in Figure 2b—when the TM polarization wave was vertically incident and exerted a pressure deformation on the sensor *X*-axis. When the sensor gap changed from 10 μm to 1 μm, the resonant frequency changed from 1.92 THz to 1.21 THz, and the maximum shift of resonant frequency per micron was 42.2%. The simulation results showed that both axes of the pressure sensor experienced a pressure sensing effect when the polarization state of the incident light was changed.

**Figure 3:**
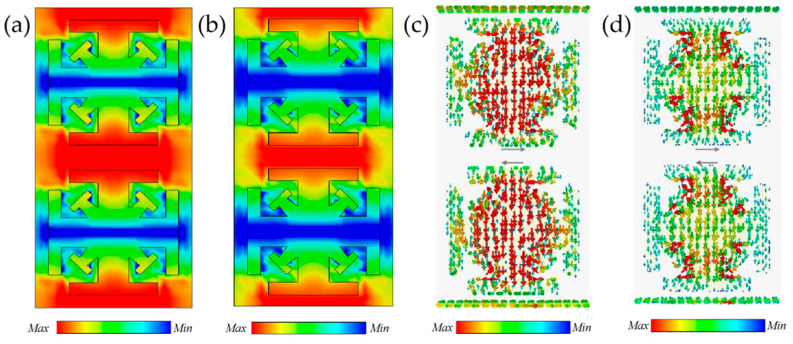
(**a**) 1.92 THz electric field map; (**b**) 1.92 THz electric field map; (**c**) 1.92 THz current profile; (**d**) 1.91 THz current profile.

In order to further understand the pressure sensing performance of the metamaterial pressure sensor, the electric field and current distribution of the device in the TM polarization state were simulated and analyzed using the CST simulation software. Figure 3a,b show the electric field distribution of the sensors at 1.92 THz and 1.91 THz, respectively, and Figure 3c,d show the current distribution.

As shown in the electric field distribution diagram, when the electromagnetic wave was incident vertically, the left and right endpoints of the double-upright cross structure were equivalent to two capacitors that concentrated the electric field on the upper and lower plates, and the surface current concentrated on the middle vertical line. The incident electric field caused a large amount of surface charge accumulation on each arm of the double-upright cross structure, and the charge formed a strong electric field between its gaps.

Metal resonators have strong dipole resonances in THz-wave polarized cells, and the resulting resonances can effectively control the permittivity. As shown in Figure 3c,d, the current was distributed on the surface of the resonance frequency. The current mainly flowed into the center from the four sides and was concentrated in the central ring region. The double-upright cross structure spacing of the electromagnetic pressure sensor changed with the pressure exerted by the outside world, and the capacitance value of the sensor changed accordingly to obtain a current resonance frequency. Therefore, it was necessary to implement a theoretical analysis of the capacitance and resonance frequency. The formula of sensor capacitance and the resonance frequency is shown in Equations (1) and (2).

(1) $$C=\frac{\varepsilon_{0}S_{0}}{4\pi k_{0}g}$$

Here, *g* is the capacitive cavity spacing; $S_{0}$ is the opposite area of two double-upright cross type structures; $\varepsilon_{0}$ is the dielectric constant of PTFE; and $k_{0}$ is the electrostatic constant. In Equation (1), the only variation is the distance between the two plates. Pressure is applied to make the distance between the two double-upright cross structures become $g_{0}$, and the capacitance value is obtained from Equation (2).

(2) $$f=\frac{1}{2\pi }\frac{1}{\sqrt{LC_{0}}}$$

where *L* represents the equivalent inductance in the equivalent circuit of the pressure sensor, which can be obtained from Equation (2); in this pressure sensor, the resonant frequency is mainly determined by the capacitance. It can be seen that the equivalent capacitance of the sensor increases as the gap decreases at different pressures, which results in a blueshift of the resonant frequency of the metamaterial structure element.

### 3.1. Sensitivity Analysis

The sensitivity of a sensor is an important indicator to measure the performance of a device. Its calculation method is shown in Equation (3):

(3) $$S=\frac{\Delta y}{\Delta x}$$

**Figure 4:**
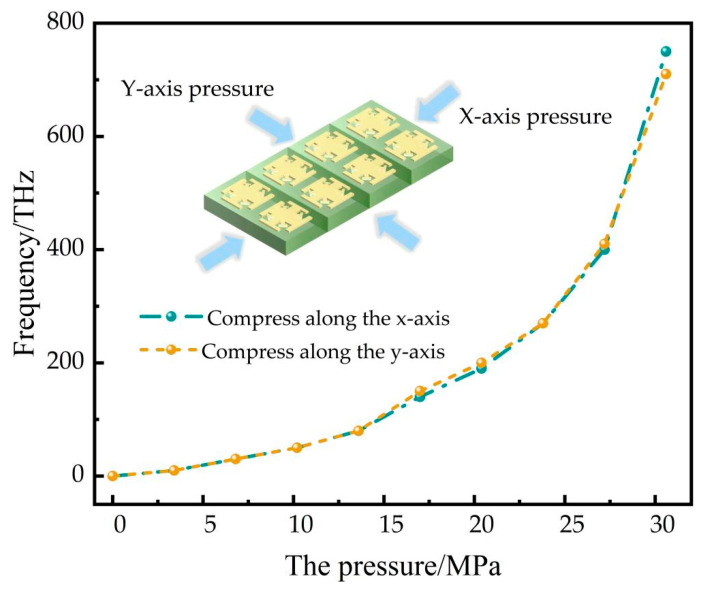
Compression biaxial resonance frequency graph.

In Equation (3), Δy represents the variation of resonant frequency and Δx represents the variation of device displacement under different pressures. Figure 4 shows the corresponding resonant frequency variation curves when pressure is applied on the *Y* and *X* axes. As can be seen from Figure 4 and Equation (2), the maximum sensitivity of the pressure sensor was 346 GHz/μm for strain along the *Y* axis in the TE polarization state, and 340 GHz/μm for strain along the *X* axis in the TM polarization state. Moreover, the spectral variation curves of the sensor’s projection coefficients in the biaxial axis were in fundamental agreement with each other, and the sensor had excellent sensing performance.

In contrast to previous studies of metamaterial THz pressure sensors, existing pressure sensors have a sensitivity of 107 GHz/um in the same frequency band [15], and the sensitivity of the sensor in this paper was increased by 3.24 times.

### 3.2. Influence Structural Parameters on Sensor

**Figure 5:**
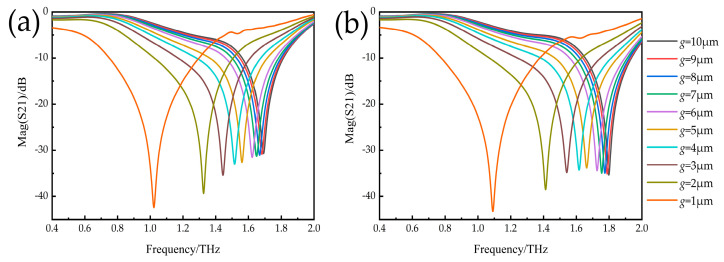
Influence of different transverse axis length k on sensing performance: (**a**) simulation results of S21 with *k* = 13 μm; (**b**) simulation results of S21 with *k* = 12 μm.

It can be seen from the electric field distribution diagram that the most crucial parameter affecting the sensing performance is the transverse axis length *k*. To obtain a better model size, this section is devoted to analyzing the effect of different values of *k* on the sensing performance. When other conditions remain unchanged, *k* values were changed to 13 μm and 12 μm, respectively. The obtained spectral variation curves of transmission coefficients in the TE polarization state are shown in Figure 5a,b.

It can be seen from Figure 5 that the maximum sensitivity of the sensor was 320 GHz/μm when *k* equaled 13 μm. When *k* decreased from 13 μm to 12 μm, the maximum sensitivity of the sensor was 300 GHz/μm. The sensitivity of the sensor gradually increased as the length of the transverse axis decreased. However, decreasing the value of *k* caused the structure to become over-compact, which is not favorable for machining. To simplify the difficulty of post-processing, this paper determined that the sensor’s transverse axis length *k* was 11 μm.

### 3.3. The Influence of the Number of Resonant Structures on the Sensor

**Figure 6:**
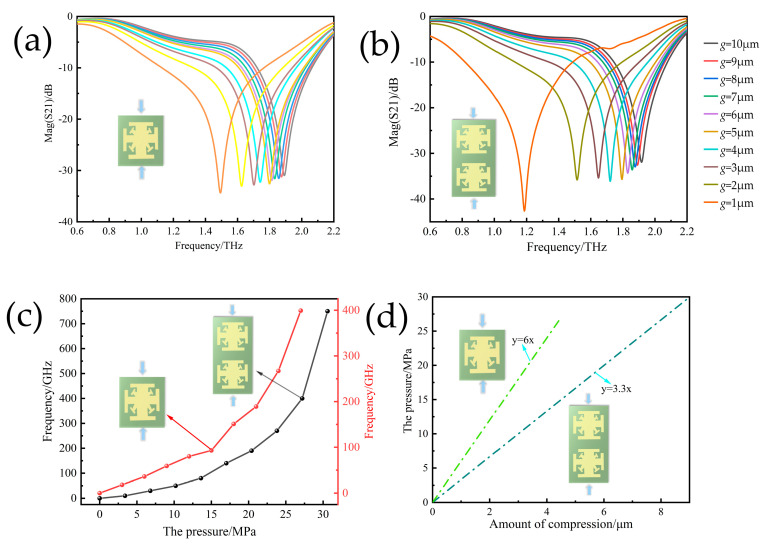
(**a**) Spectrum variation curve of a single double-upright cross font; (**b**) spectrum variation curve of a double-upright cross font; (**c**) curve of resonant frequency variation of different structures; (**d**) pressure-displacement curves of different structures.

Currently, pressure sensors of the same material are analyzed using only the dual structure as the fundamental unit. It remains to be investigated whether the individual structures have pressure-sensing effects. In this regard, electromagnetic simulations of single and double structures were performed and the respective pressure ranges were measured in COMSOL Multiphysics. The concrete results are shown in Figure 6.

Figure 6a,b show the S-parameter spectrum variation curves of a single double-upright cross font structure and a symmetrical double-upright cross font structure with pressure changes, while Figure 6c shows the resonance frequency variation curves of two different structures with pressure changes. By comparison, it was found that the basic unit of the sensor composed of a symmetrical double-upright cross font had the advantages of faster curve change and higher sensitivity than those of a sensor composed of a single meter font.

Figure 6d shows the pressure and displacement dependence of the pressure sensor under the COMSOL Multiphysics simulation. The two sensor structures designed were converted to STL file format and imported into COMSOL Multiphysics to add the base material polytetrafluoroethylene and the resonant unit gold materials from the material library. From the simulations, it can be concluded that the maximum measurement range was up to 30 MPa for symmetric metronome structures and up to 27 MPa for single metronomes. Therefore, considering the sensor pressure measuring range and sensitivity, the choice of a symmetrical double-upright cross font structure is more in line with the demand in practical application.

### 3.4. The Effect of Angle of Incidence on Sensor Performance

**Figure 7:**
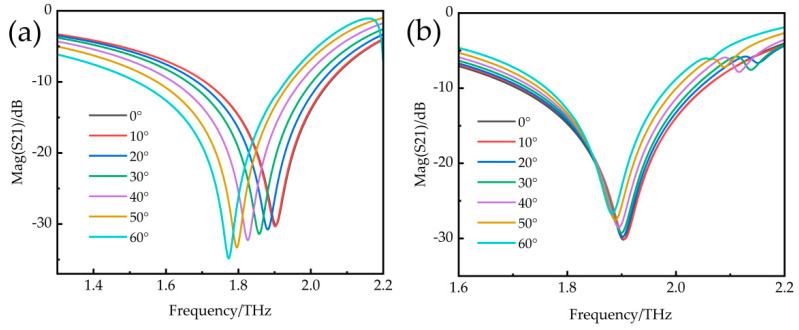
(**a**) Influence of incident angle on S21 in TE polarization state; (**b**) influence of incident angle on S21 in TM polarization state.

The above performance results were based on the positive incidence of THz waves at the pressure sensor surface. To further analyze its application value, the incidence angle of the THz wave at the sensor surface was varied, and the connection between incidence angle and reflector reflection performance in different polarization states is discussed. As shown in Figure 7, in the TE polarization regime, when no pressure was applied at this time, the sensor transmission spectrum gradually shifted to the left as the incident angle gradually increased. Within 0–10°, there was virtually no effect on the sensor itself, and when the incident angle was greater than 10°, the resonant frequency red shift gradually increased and the transmission coefficient gradually increased. In the TM polarization regime, the transmission spectrum of the sensor gradually shifted red with the increasing incident angle, but the shift range was narrow and the transmission coefficient gradually decreased. The reason for this was that in the TM polarization state, the magnetic field at different incident angles of the sensor remained constant in the y-direction and was always able to produce an effective magnetic response, while in the TE polarization state, as the y-component of the incident magnetic field became smaller and smaller, the coupling between it and the metamaterial structure became weaker.

### 3.5. Device Fabrication

**Figure 8:**
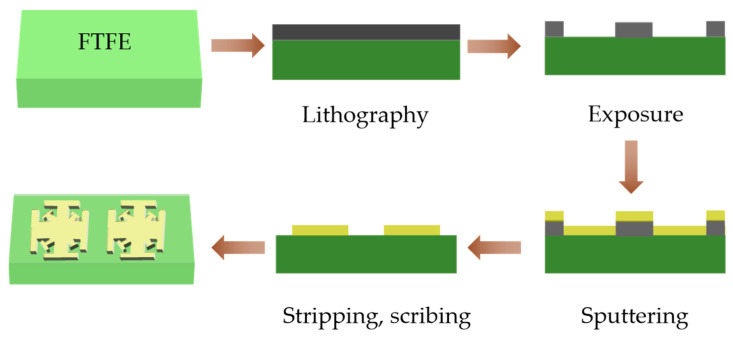
Flow chart of pressure sensor preparation.

The detailed fabrication steps, as shown in Figure 8, are provided in this section. In this paper, polymer chemistry was used for the preparation. In the first step, liquid fluorine and chlorine gas were passed into the reactor and then initiators, such as hydrogen peroxide, were added to form radicals that broke the double bonds in the tetrafluoroethylene molecule and gradually polymerized into PTFE. The PTFE obtained by polymerization in the second step appeared as white granules, which needed to be crushed by mechanical grinding. The purpose of this step was to grind the PTFE particles obtained by polymerization into a uniform fine powder. In the third step, the ground state PTFE was granulated to obtain PTFE granules with different particle sizes. This also facilitated the production of different types and shapes of PTFE products. In general, granulated PTFE pellets were also subjected to a coating process to meet the final application requirements. In the fourth step, the PTFE was processed and shaped by a pressing process. The PTFE pellets were added to a die and pressed at a certain temperature and pressure. This allowed the PTFE pellets to be formed by high-temperature thermal pressing and the PTFE substrate to be cleaned, as shown in Figure 8. In the fifth step, the substrate was lithographed by a lithography machine and dried before being developed. In the sixth step, the developed substrate was sputtered with 50 nm chromium as an adhesion layer and 0.2 um of gold thickness. In the seventh step, the sputtered sensor was peeled and scraped, and the pressure sensor designed in this paper was finally fabricated.

In Table 3, the performance of the proposed sensor architecture is compared with that of other sensors [17,20,21,22,23,24]. It was found that within the same terahertz band, the range and sensitivity of the sensor proposed were higher than those in the literature [6]. Compared with microwave phase material pressure sensors, the proposed metamaterial pressure sensor has significant advantages in terms of manufacturing costs, sensitivity, and measurement range.

## 4. Conclusions

This paper presented a double-upright cross type pressure sensor based on metamaterial. A THz metamaterial pressure sensor with a sensitivity 3.24 times that of existing pressure sensors [6] was obtained by analyzing the structural parameters of polarization strain under different polarization states. In addition to generating strain in both axes, this sensor has the advantage of being simple in construction and low in cost. At the same time, the sensor base is made of high temperature resistance and corrosion resistance material, so that it can work properly in the range of 200 °C. The proposed metamaterial pressure sensor has promising applications in special contexts. In future studies, it will still be necessary to continuously optimize the structure and explore different materials to further improve the sensitivity of the sensor.

## Figures and Tables

**Table 1:** Detailed parameters of metal rice type structure.

Parameters	*c*	*d*	*e*	*k*	*m*	*n*	*o*	*p*
Value/μm	34	5	10	11	5	2.8	60	50

**Table 2:** The resonant frequency decreases with spacing.

Spacing g/um	10	9	8	7	6	5	4	3	2	1
Change in resonant frequency/GHz	0	10	30	50	79	138	191	269	404	750

**Table 3:** The proposed pressure sensor compared with the reference.

Ref	Sensor Materials	Operating Pressure	Sensitivity	Cost
[17]	PDMS, Au	0–150 kpa	107 GHz/μm	Moderated
[20]	Resin, Ag ink	0–3.54 kPa	1.13 × 10^5^ kHz/kPa	Moderated
[21]	SU-8, Au	0–60 mmHg	1.083 MHz/mmHg in air, 683 kHz/mmHg in saline, 120 kHz/mmHg in air	High
[22]	Polyimide, Au	0–213.3 kPa	11.25 kHz/kPa	Moderated
[23]	Si, SiO_2_, Au, Pyrex, Polyimide, Steel	0–50 mmHg	15 kHz/mmHg	High
[24]	Biodegradable Polymer, Zn/Fe bilayer conducto	0–20 kPa	39 kHz/kPa	High
Proposed	Teflon, gold	0–30 MPa	346 GHz/μm	Low

## Data Availability

The data that support the findings of this study are available from the corresponding author upon reasonable request.
